# Prognostic and diagnostic value of circRNA expression in colorectal carcinoma: a meta-analysis

**DOI:** 10.1186/s12885-020-06932-z

**Published:** 2020-05-19

**Authors:** Jinpeng Yuan, Dongming Guo, Xinxin Li, Juntian Chen

**Affiliations:** Department of Gastrointestinal Surgery, the First Affiliated Hospital of Shantou University Medical College, Shantou, China

**Keywords:** Circular RNA, Colorectal cancer, Diagnosis, Prognosis

## Abstract

**Background:**

Circular RNAs (circRNAs) are research hotspots in the network of noncoding RNAs in numerous tumours. The purpose of our study was to evaluate the clinicopathological, prognostic and diagnostic value of circRNAs in colorectal cancer.

**Methods:**

The PubMed, Cochrane Library, and Web of Science online databases were searched for relevant studies before May 15, 2019. Pooled hazard ratios (HRs) and odds ratios (ORs) with 95% confidence intervals (CIs) were calculated to assess the association between circRNAs expression, and overall survival (OS) and clinical parameters. Pooled sensitivity, specificity, and the area under the curve (AUC) were employed to assess the diagnostic value of circRNAs.

**Results:**

A total of 19 studies were enrolled in this meta-analysis, with 11 on clinicopathological parameters, 8 on prognosis and 7 on diagnosis. For clinicopathological and prognostic value, elevated expression of oncogenic circRNAs was correlated with poor clinical parameters (tumor size: OR = 1.769, 95% CI: 1.097–2.852; differentiation grade: OR = 1.743, 95% CI: 1.032–2.946; TNM stage: OR = 3.320, 95% CI: 1.529–7.207; T classification: OR = 3.410, 95% CI: 2.088–5.567; lymph node metastasis: OR = 3.357, 95% CI: 2.160–5.215; distal metastasis: OR = 4.338, 95% CI: 2.503–7.520) and worse prognosis (HR = 2.29, 95% CI: 1.50–3.52). However, elevated expression of tumor-suppressor circRNAs was correlated with better clinical parameters (differentiation grade: OR = 0.453, 95% CI: 0.261–0.787; T classification: OR = 0.553, 95% CI: 0.328–0.934; distal metastasis: OR = 0.196, 95% CI: 0.077–0.498) and favorable prognosis (HR = 0.37, 95% CI: 0.22–0.64). For diagnostic value, the pooled sensitivity, specificity, and AUC were 0.82 (95% CI, 0.75–0.88), 0.72 (95% CI, 0.66–0.78), and 0.82 (95% CI, 0.78–0.85), respectively.

**Conclusions:**

These results indicate that circRNAs may be potential biomarkers for the diagnosis and prognosis of colorectal cancer.

## Background

Circular RNAs (circRNAs), consisting of a circular configuration through a typical 5′ to 3′-phosphodiester bonds, are a novel class of endogenous noncoding RNAs [1–3]. CircRNAs play a special role as molecular markers in many human diseases including tumors, due to their conservation, abundance and tissue specificity [4]. In addition, circRNAs can be classified into four categories: exon circRNAs, intron circRNAs, exon-intron circRNAs, and intergenic circRNAs [5]. Different types of circRNAs have distinct functions, including interacting with RNA binding proteins, regulating the stability of the mRNAs, regulating gene transcription, sponging microRNAs and participating in translation [5–7]. However, the underlying mechanisms and functions of circRNAs remain uncertain.

Extensive studies have indicated that circRNAs play a major role in tumorigenesis, the development of cardiovascular diseases, and the pathogenesis of neurodegenerative diseases [8]. However, the differential expression of circRNAs and their definite functions are still not totally clear in colorectal cancer (CRC). Colorectal cancer is among the most common malignancies of the digestive system and the fourth leading cause of cancer-related death worldwide [9]. Although considerable progress has been made in the diagnosis and treatment of this disease, the prognosis of CRC patients is still poor, due to the delay in early diagnosis and the high frequency of metastasis and recurrence [10]. In this study, we performed a meta-analysis and a comprehensive search of all relevant literature to summarize the diagnostic, prognostic, and clinical significance of circRNAs in CRC.

## Methods

### Data search strategy

The PubMed, Cochrane Library, and Web of Science online databases were searched for studies on circRNA research that were published in English before May 15, 2019. The following search strategy was applied: (1) “circRNA” or “circular RNA” and (2) “colorectal cancer” or “colorectal carcinoma” or “colorectal tumour” or “CRC”. Two researchers (JPY and DMG) assessed the title, abstract and full text to identify the appropriate articles. Other researchers (XXL), together with two researchers (JPY and DMG) were involved in the data extraction. Any disagreements were settled by a third researcher (JTC). Then, the data were extracted from the selected articles and populated it into a table.

### Inclusion and exclusion criteria

This study used the following criteria when selecting articles. Studies that met the following inclusion criteria were included in the meta-analysis: (1) patients with a pathological diagnosis of CRC; (2) cohort study or case-control study; and (3) studies that detected the circRNA expression level and provided information on the clinicopathological features and prognosis of patients. Studies were excluded if the following excluded criteria were met: (1) studies irrelevant to CRC or circRNAs; (2) data similar to that in prior studies; (3) case reports, letters, animal experiments, reviews, conference reports and meta-analysis; and (4) insufficient data.

### Data extraction and quality assessment

All relevant studies were independently screened by two researchers (JPY and DMG) and the following data were extracted from eligible studies: (1) first author, publication year, type of cancer and circRNA, sample size and detection method of circRNA; (2) the role of circRNAs, follow-up time; (3) diagnostic sensitivity and specificity of circRNAs; and (4) clinicopathological features with age, gender, tumour size, tumor location, differentiation grade, TNM stage, T classification, lymph node metastasis, distal metastasis [11]. The Newcastle-Ottawa Scale (NOS) [12] was adopted for the quality assessment of the studies by two independent researchers (JPY and DMG). A third investigator (XXL) discussed any differences. A study with a score ≥ 7 was considered of high quality.

### Statistical analysis

Statistical analysis was conducted using STATA software (version 14). Pooled ORs and 95% CIs were used to explore the association between circRNAs expression and clinicopathological features. HRs and 95% CIs were used to assess the prognostic value of circRNAs. The number of true positive (TP), false positive (FP), false negative (FN) and true negative (TN) were calculated and finally the pooled sensitivity, specificity and AUC were obtained to assess the diagnostic value of circRNAs. The chi-square test were used to evaluate heterogeneity. When the I^2^ value was < 50%, no observable heterogeneity was suggested and a fixed effects model was used [13]; otherwise, a random effects model was utilized. Sensitivity analysis was performed to explore the source of heterogeneity. Qualitative analysis of publication bias was conducted using funnel plots and quantitative analysis was conducted using Begg and Egger’s tests.

## Results

### Search results

**Fig. 1:**
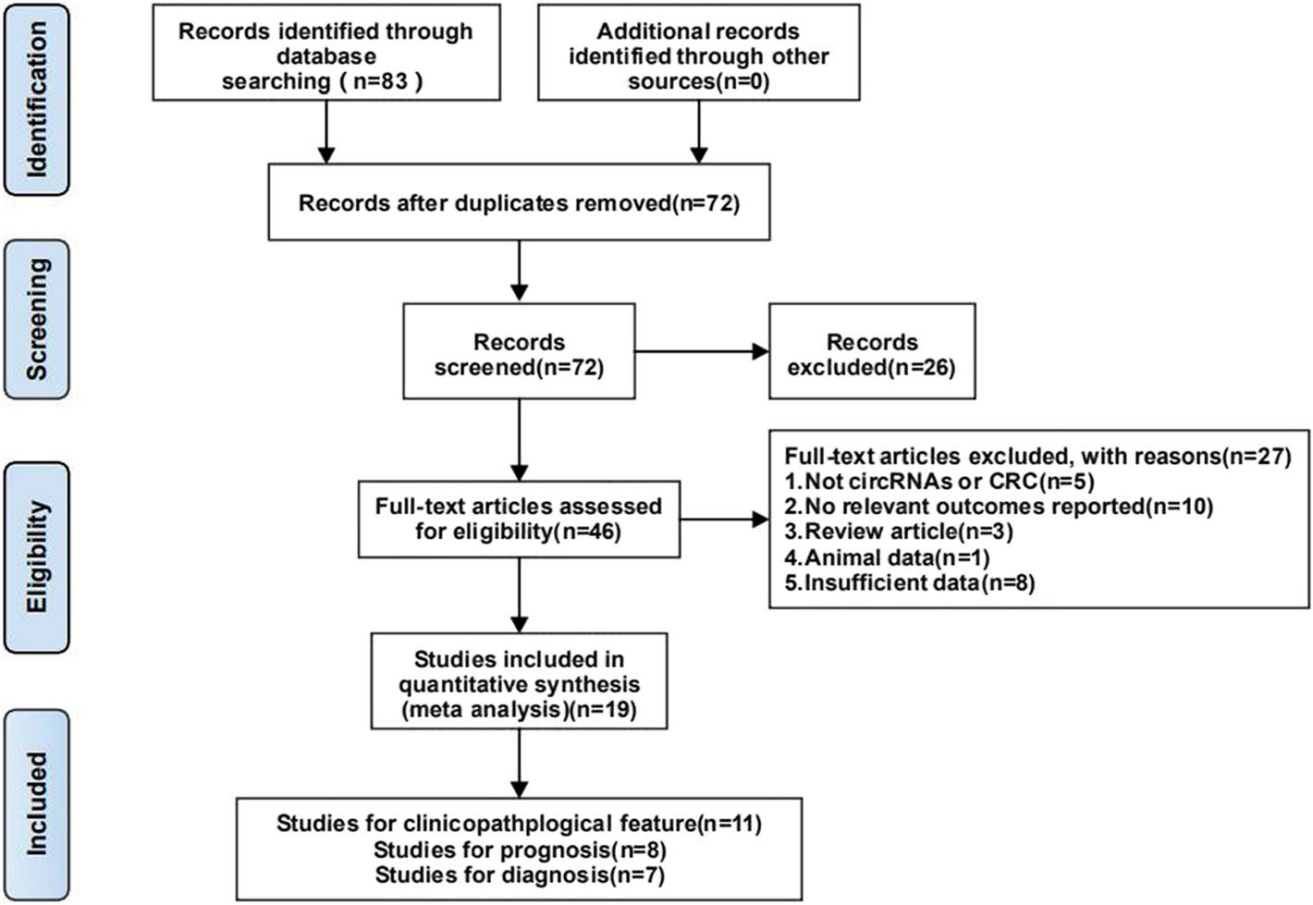
Flowchart of trial selection

As shown in Fig. 1, 83 relevant studies were obtained from several databases. After abstract reviews, 46 studies were obtained for further full-text reviews. Then, 27 articles were excluded for the following reasons: 5 were not about circRNAs or CRC, 10 did not report relevant results, 3 were review articles, 1 was animal data, and 8 had insufficient data. In summary, there were 19 studies [14–32] included in this study, with a total of 1307 patients, including 11 on clinicopathological features, 8 on prognosis and 7 on diagnosis.

### Study characteristics

The basic information of studies are showed in Table 1 and Table 2. All studies were published between 2015 and 2019. The follow-up time of patients ranged from 57 months to 123 months and the number of samples ranged from 40 to 204. As shown in Tables 1, 6 circRNAs were identified as tumour promoters, and 2 circRNAs were identified as tumour suppressors. As shown in Tables 2, 7 articles with AUC, sensitivity and specificity were included for the diagnosis analysis. The included studies were of high quality (See Supplementary Table 1, Additional File 1).

**Table 1:** Basic features of studies for prognosis analysis

				CircRNA expression				
Study	Year	CircRNA	Cancer Type	High	Low	Detection Method	Regulation	Follow-up (months)
Zeng et al. [27]	2018	circHIPK3	CRC	89	89	qRT-PCR	Upregulated	91
Fang et al. [14]	2018	circ_100290	CRC	24	20	qRT-PCR	Upregulated	59
Weng et al. [31]	2017	circCiRS7	CRC	89	76	qRT-PCR	Upregulated	123
Wang et al. [25]	2019	circPVT1	CRC	32	32	qRT-PCR	Upregulated	58
Jin et al. [17]	2018	circ_0136666	CRC	26	26	qRT-PCR	Upregulated	60
Wang et al. [26]	2018	circ_0071589	CRC	20	20	qRT-PCR	Upregulated	58
Li et al. [18]	2018	circ_0000711	CRC	50	51	qRT-PCR	Downregulated	60
Wang et al. [23]	2018	circ_0014717	CRC	23	23	qRT-PCR	Downregulated	57

*CRC* Colorectal cancer; *qRT-PCR* Quantitative real time polymerase chain reaction

**Table 2:** Basic features of studies for diagnosis analysis

				Sample size				Diagnosis power		
Study	Year	CircRNA	Cancer Type	case	control	Method	Regulation	Sen.	Spe.	AUC.
Ji et al. [16]	2018	circ_0001649	CRC	64	64	qRT-PCR	downregulated	0.828	0.781	0.857
Li et al. [19]	2018	circITGA7	CRC	69	48	qRT-PCR	downregulated	0.928	0.667	0.879
Wang et al. [24]	2017	circ_0000567	CRC	102	102	qRT-PCR	downregulated	0.833	0.765	0.865
Zhuo et al. [28]	2017	circ_0003906	CRC	122	40	qRT-PCR	downregulated	0.803	0.725	0.818
Ruan et al. [22]	2019	circ_0002138	CRC	35	35	qRT-PCR	downregulated	0.629	0.743	0.725
Wang et al. [32]	2015	circ_001988	CRC	31	31	qRT-PCR	downregulated	0.680	0.730	0.788
Li et al. [18]	2018	circ_0000711	CRC	101	101	qRT-PCR	downregulated	0.910	0.58	0.810

*AUC* Area under the ROC curve; *qRT-PCR* Quantitative real-time polymerase chain reaction; *Sen* Sensitivity; *Spe.* Specificity; *CRC* Colorectal cancer

### Clinicopathological parameters

The associations between circRNAs and the clinical parameters are shown in Table 3. Up-regulation of oncogenic circRNAs was closely associated with unfavorable clinical features (tumor size: OR = 1.769, 95% CI: 1.097–2.852; differentiation grade: OR = 1.743, 95% CI: 1.032–2.946; TNM stage: OR = 3.320, 95% CI: 1.529–7.207; T classification: OR = 3.410, 95% CI: 2.088–5.567; lymph node metastasis: OR = 3.357, 95% CI: 2.160–5.215; distal metastasis: OR = 4.338, 95% CI: 2.503–7.520). Additionally, down-regulation of tumor-suppressor circRNAs was closely associated with favorable clinical parameters (differentiation grade: OR = 0.453, 95% CI: 0.261–0.787; T classification: OR = 0.553, 95% CI: 0.328–0.934; distal metastasis: OR = 0.196, 95% CI: 0.077–0.498). However, there was no difference between oncogenic circRNAs expression and other clinical parameters such as age, gender, and tumor location.

**Table 3:** Clinical Parameters of circRNAs in CRC

	Tumor promoter			Tumor Suppressor		
	OR	95%CI	P	OR	95%CI	P
Age (older/younger)	1.078	0.737–1.577	0.698	0.589	0.241–1.437	0.224
Gender (M/W)	1.114	0.757–1.639	0.968	0.805	0.491–1.320	0.390
Tumor size (larger/smaller)	1.769	1.097–2.852	0.019	0.658	0.382–1.132	0.131
Tumor location (rectum/colon)	0.888	0.572–1.380	0.598	0.902	0.480–1.694	0.748
Differentiation grade (poor/well & moderate)	1.743	1.032–2.946	0.038	0.453	0.261–0.787	0.005
TNM stage (III + IV/I + II)	3.320	1.529–7.207	0.002	0.442	0.187–1.042	0.062
T classification (T3 + T4/T1 + T2)	3.410	2.088–5.567	0.000	0.533	0.328–0.934	0.027
Lymph node metastasis (Y/N)	3.357	2.160–5.215	0.000	0.389	0.116–1.307	0.127
Distant metastasis (Y/N)	4.338	2.503–7.520	0.000	0.196	0.077–0.498	0.001

*CI* Confidence interval; *M* Men; *N* No; *W* Women; *Y* Yes; *OR* Odds ratio. The results are in bold if *p* < 0.05

### Overall survival

**Fig. 2:**
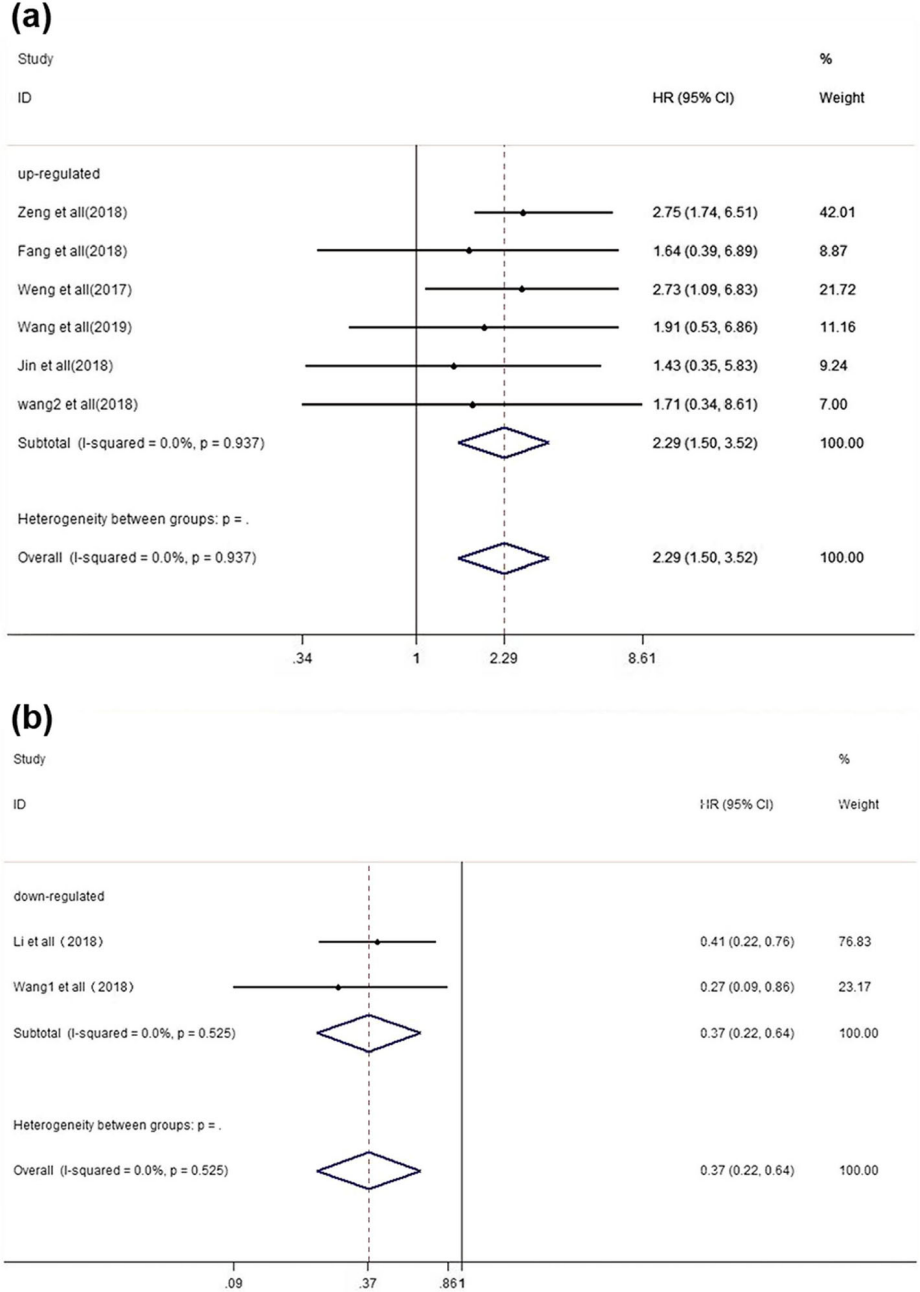
Forest plots for the association between circRNAs and overall survival (OS) in colorectal cancer (CRC). **a** oncogenic circRNAs; **b** tumor suppressor circRNAs

Up-regulation of oncogenic circRNAs was notably associated with worse prognosis (HR = 2.29, 95% Cl: 1.50–3.52, *p* < 0.001, Fig. 2 a), and a fixed-effects model was utilized as no heterogeneity was found (I^2^ = 0.0%, *p* = 0.937). In addition, down-regulation of tumour-suppressor circRNAs was associated with better prognosis (HR = 0.37, 95% Cl: 0.22–0.64, *p* < 0.001, Fig. 2 b), and a fixed-effects model was applied because of no heterogeneity between studies (I^2^ = 0.0%, *p* = 0.525).

### Diagnosis analysis

**Fig. 3:**
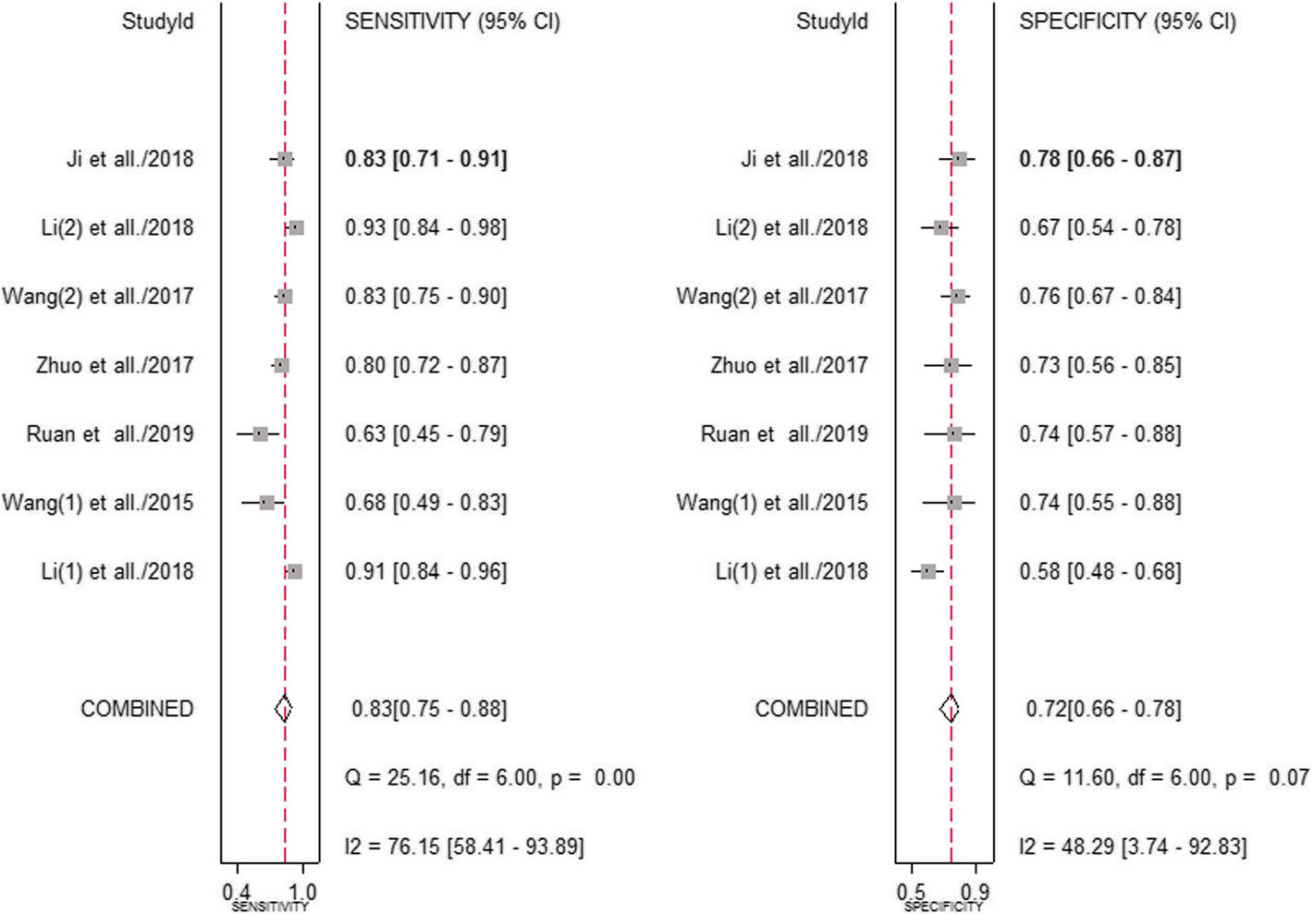
Forest plots for the pooled sensitivity and specificity of circRNAs

**Fig. 4:**
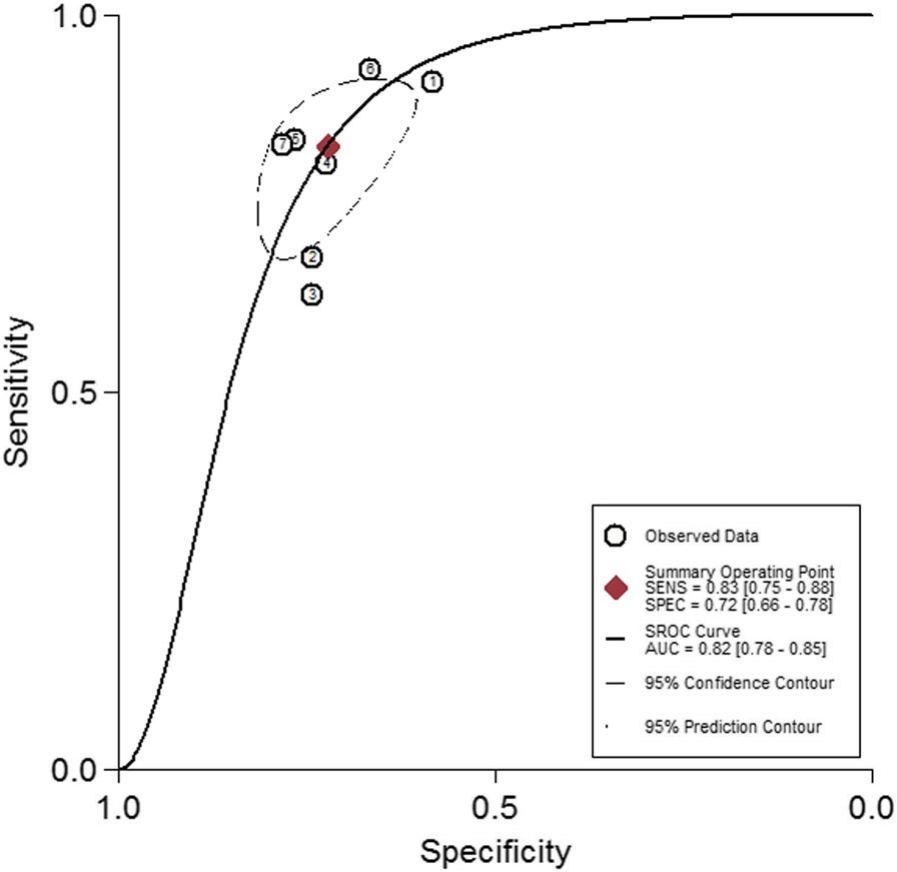
SROC curve in the diagnostic analysis

To further evaluate the diagnostic value of circRNAs, the pooled sensitivity and specificity were calculated, and the results were shown in Fig. 3. And a random-effects model was utilized because of high heterogeneity between studies (I^2^ = 76.15% and I^2^ = 48.29%). The pooled results showed a sensitivity of 0.83 (95% CI: 0.75–0.88) and a specificity of 0.72 (95% CI: 0.66–0.78). In addition, the summary receiver operator characteristic (SROC) curve analysis indicated AUC of 0.82 (95% CI 0.78–0.85, Fig. 4). Taken together, these results suggested that circRNAs have a good diagnostic accuracy for CRC.

### Publication bias and sensitivity analysis

No evidence of publication bias were identified from the funnel plot by qualitative analysis (See Supplementary Fig. 1, Additional File 2). In quantitative analysis, there was no obvious publication bias by Begg’s (*p* = 0.213, See Supplementary Fig. 2, Additional File 2) and Egger’s test (*p* = 0.722, See Supplementary Fig. 3, Additional File 2). Furthermore, Deek’s funnel plot asymmetry test [33] was performed to assess the publication bias among studies for diagnosis analysis, and the result showed no obvious publication bias was found (*p* = 0.07, See Supplementary Fig. 4, Additional File 2). Sensitivity analysis indicated the pooled results were stable in our studies (See Supplementary Fig. 5, Additional File 2).

## Discussion

Recently, many studies have focused on the significant role of circRNAs, whereas no relevant meta-analyses on circRNA expression in CRC have been performed. A total of 1307 cancer patients from 19 eligible studies were collected and analyzed in this study, including 7 on diagnosis, 8 on prognosis, and 11 on clinicopathological features. For diagnostic value, the summarized results revealed AUC of 0.82, with a sensitivity of 83% and a specificity of 72%. For clinical and prognostic value, abnormal expression of circRNAs were closely associated with clinical parameters and prognosis.

Our current study observed a significant relationship between abnormal circRNA expression and its diagnostic value in CRC patients. As aberrant expression of circRNAs in different tumor tissue can be easily detected, measurements can be performed conveniently and economically. Coupled with the structural stability of circRNAs, circRNAs are considered as potential biomarkers for the diagnosis of CRC patients. Although sensitivity analysis showed no significant heterogeneity, more pertinent investigations are warranted to corroborate our findings.

In previous meta-analyses, only five meta-analyses [34–38] detected an association between the circRNAs and carcinoma. However, in the studies of Wang et al. [34], Chen et al. [35] and Li et al. [36], only one study was included to investigate the relationship between the circRNAs and CRC. Li et al. [37] and Ding et al. [38] assessed the diagnostic value of circRNAs for human cancers, in which five articles were included to investigate the diagnostic value of circRNAs in CRC, whereas they failed to discuss the role of circRNAs in CRC patients. In the present study, we collected all the relevant articles published to date and performed a meta-analysis including 19 articles with 1307 CRC patients. Furthermore, we evaluated the prognostic and diagnostic value of circRNA expression in CRC patients. Nonetheless, further large-scale studies are needed to confirm these results.

However, several limitations must be considered when interpreting the conclusions of this meta-analysis. First, since all patients included in the article were from China, this reduced the applicability of the results across different ethnicities and regions. Moreover, there was a limited number of articles for a subgroup analysis. Furthermore, a relatively small number of patients was included in this meta-analysis, so larger-scale studies would be necessary to verify the obtained results. Finally, several studies did not provide HRs with their 95% CIs in the article, so we needed to extract them from the Kaplan-Meier survival curve.

## Conclusions

In summary, our study demonstrated a crucial relationship between the aberrant expression of circRNAs and clinicopathological, prognostic, and diagnostic value in CRC patients. Furthermore, circRNAs may be promising biomarkers and treatment targets for colorectal cancer.

## Supplementary information

**Additional file 1: Table S1.** Quality assessment of included studies (Newcastle-Ottawa Scale).

**Additional file 2: Figure S1.** Funnel plot for the evaluation of publication bias. **Figure S2.** Begg’s funnel plot for the evaluation of publication bias. **Figure S3.** Egger’s funnel plot for the evaluation of publication bias. **Figure S4.** Deeks’ funnel plot asymmetry test for the evaluation of publication bias. **Figure S5.** Sensitivity analysis to assess the stability of results.

## Data Availability

All data analyzed during this study are included in this article.

## Abbreviations

OR: Odds ratios
95% CI: 95% Confidence interval
HR: Hazard ratio
OS: Overall survival
circRNAs: Circular RNAs
CRC: Colorectal cancer
SROC: The summary receiver operator characteristic curve
AUC: The area under the curve
