# Supplementary material for: Prognostic and diagnostic value of circRNA expression in colorectal carcinoma: a meta-analysis
Source: BMC Cancer. 2020 May 19;20:448. doi: 10.1186/s12885-020-06932-z (PMC7238613; doi:10.1186/s12885-020-06932-z)

**Supplementary Table 1. Quality assessment of included studies (Newcastle-Ottawa Scale).**

| **Study** | **Selection** | | | **Comparability** | | | **Outcome** | | **Total** |
| --- | --- | --- | --- | --- | --- | --- | --- | --- | --- |
|  | **Adequacy of case definition** | **Number**  **of case** | **Representativeness of the cases** | **Ascertainment of**  **relevant cancers** | **Ascertainment of**  **detection method** | **CircRNA expression** | **Assessment of outcome** | **Adequate**  **follow up** |  |
| Li et al. | 1 | 1 | 1 | 1 | 1 | 1 | 1 | 1 | 8 |
| Wang et al. | 1 | 1 | 1 | 1 | 1 | 1 | 1 | 1 | 8 |
| Wang et al. | 1 | 1 | 1 | 1 | 1 | 1 | 1 | 0 | 7 |
| Jin et al. | 1 | 1 | 1 | 1 | 1 | 1 | 1 | 1 | 8 |
| Zeng et al. | 1 | 1 | 1 | 1 | 1 | 1 | 1 | 1 | 8 |
| Wang et al. | 1 | 0 | 1 | 1 | 1 | 1 | 1 | 1 | 7 |
| Fang et al. | 1 | 1 | 1 | 1 | 1 | 1 | 1 | 1 | 8 |
| Weng et al. | 1 | 1 | 1 | 1 | 1 | 1 | 1 | 1 | 8 |
| Wang et al. | 1 | 1 | 1 | 1 | 1 | 1 | 1 | 0 | 7 |
| Ji et al. | 1 | 1 | 1 | 1 | 1 | 1 | 1 | 0 | 7 |
| Wang et al. | 1 | 1 | 1 | 1 | 1 | 1 | 1 | 0 | 7 |
| Ruan et al. | 1 | 1 | 1 | 1 | 1 | 1 | 1 | 0 | 7 |
| Li et al. | 1 | 1 | 1 | 1 | 1 | 1 | 1 | 0 | 7 |
| Li et al. | 1 | 1 | 1 | 1 | 1 | 1 | 1 | 0 | 7 |
| Guo et al. | 1 | 1 | 1 | 1 | 1 | 1 | 1 | 0 | 7 |
| Zhang et al. | 1 | 1 | 1 | 1 | 1 | 1 | 1 | 0 | 7 |
| Xie et al. | 1 | 1 | 1 | 1 | 1 | 1 | 1 | 0 | 7 |
| Li et al. | 1 | 1 | 1 | 1 | 1 | 1 | 1 | 0 | 7 |


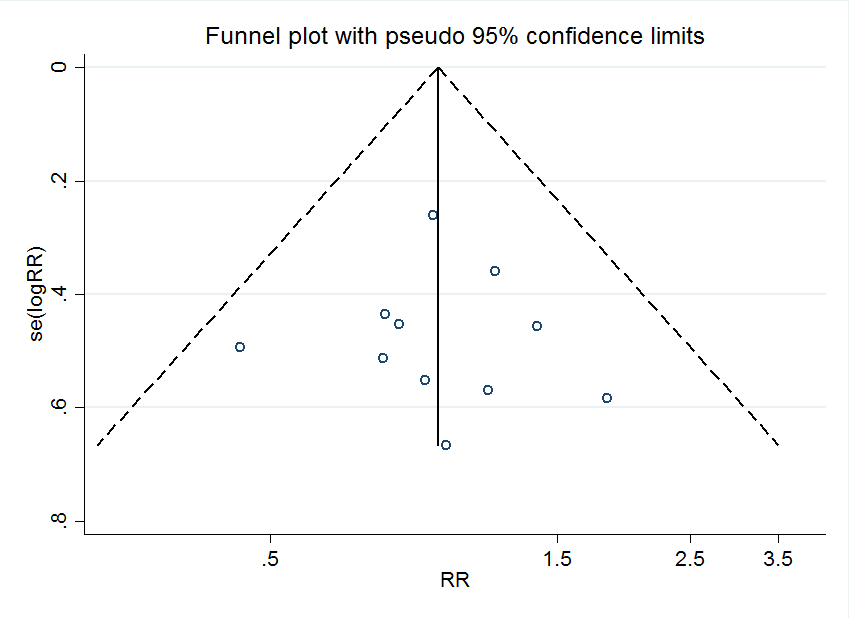

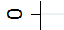


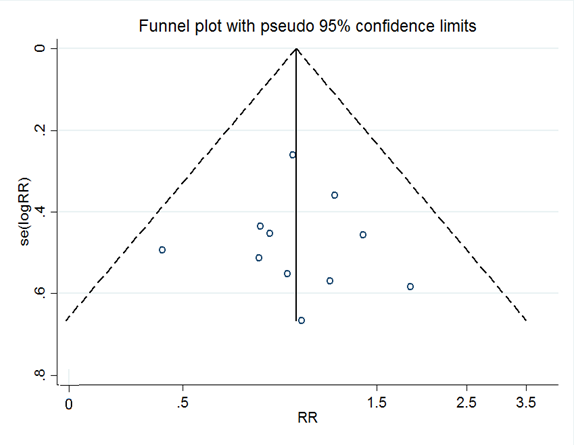

Supplement: Supplementary file 1 — Additional file 1: Table S1. Quality assessment of included studies (Newcastle-Ottawa Scale). [file 12885_2020_6932_MOESM1_ESM.doc]
