# Supplementary material for: Prognostic and diagnostic value of circRNA expression in colorectal carcinoma: a meta-analysis
Source: BMC Cancer. 2020 May 19;20:448. doi: 10.1186/s12885-020-06932-z (PMC7238613; doi:10.1186/s12885-020-06932-z)

**Supplementary Figure 1: Funnel plot for the evaluation of publication bias.**


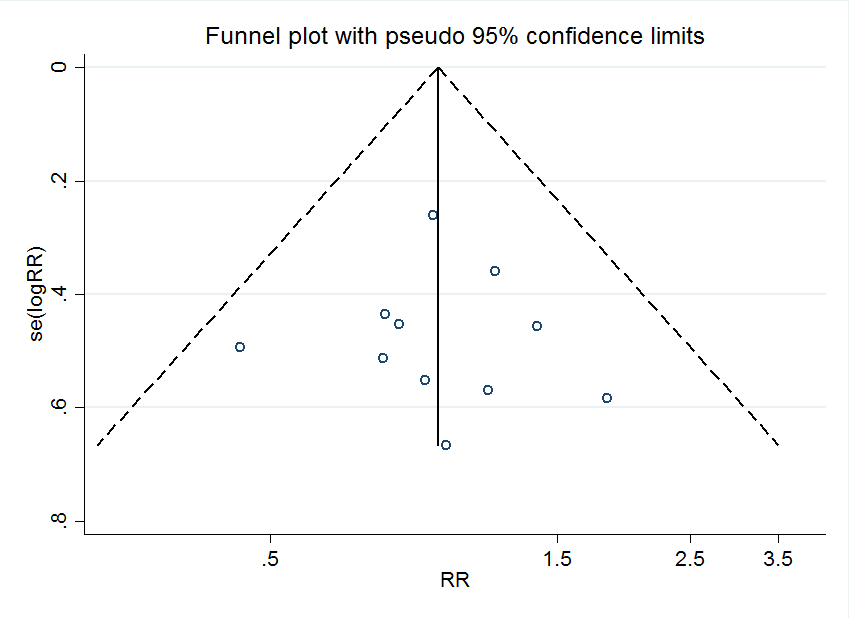

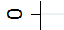


**Supplementary Figure 2: Begg’s funnel plot for the evaluation of publication bias.**


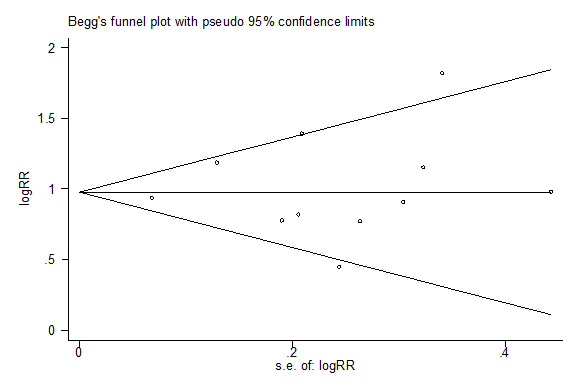


**Supplementary Figure 3:** **Egger’s funnel plot for the evaluation of publication bias.**


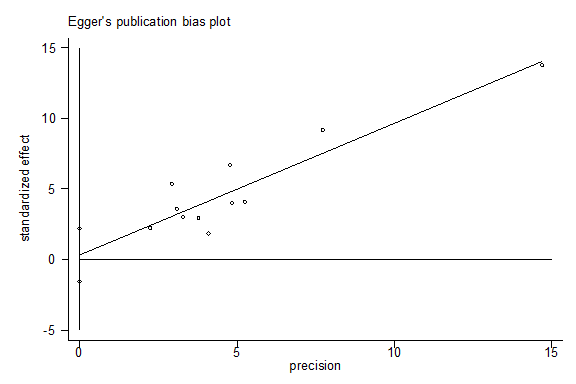


**Supplementary Figure 4: Deeks’ funnel plot asymmetry test for the evaluation of**

**publication bias.**


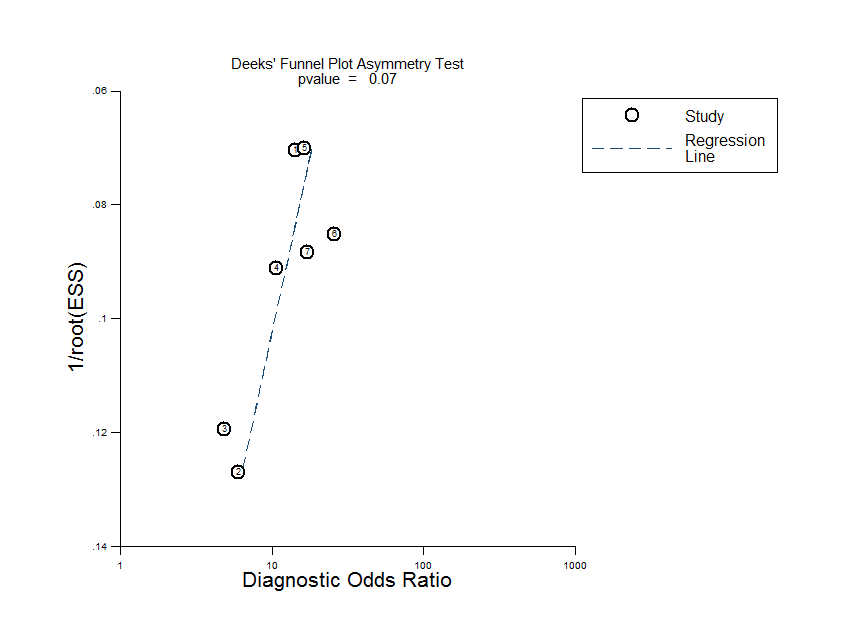


**Supplementary Figure 5: Sensitivity analysis to assess the stability of results.**


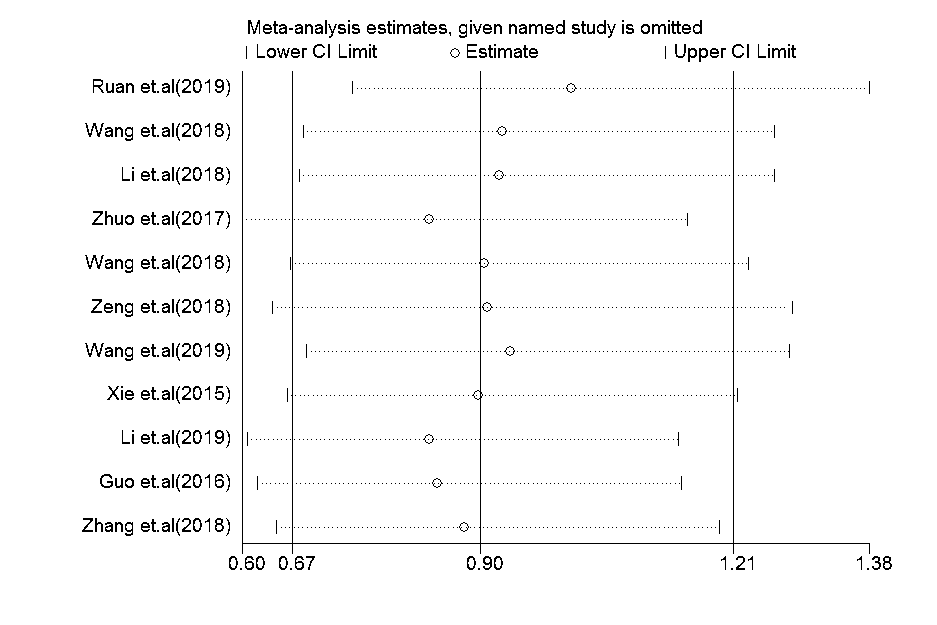

Supplement: Supplementary file 2 — Additional file 2: Figure S1. Funnel plot for the evaluation of publication bias. Figure S2. Begg’s funnel plot for the evaluation of publication bias. Figure S3. Egger’s funnel plot for the evaluation of publication bias. Figure S4. Deeks’ funnel plot asymmetry test for the evaluation of publication bias. Figure S5. Sensitivity analysis to assess the stability of results. [file 12885_2020_6932_MOESM2_ESM.doc]
